# 2173 RNA-nanoparticles to enhance and track dendritic cell migration

**DOI:** 10.1017/cts.2018.117

**Published:** 2018-11-21

**Authors:** Adam J. Grippin, Elias J. Sayour, Brandon Wummer, Adam Monsalve, Tyler Wildes, Kyle Dyson, Jon Dobson, Duane A. Mitchell

**Affiliations:** 1 Department of Neurosurgery, Preston A. Wells, Jr. Center for Brain Tumor Therapy, University of Florida, Gainesville, FL, USA; 2 J. Crayton Pruitt Family Department of Biomedical Engineering, University of Florida, Gainesville, FL, USA; 3 Department of Materials Science & Engineering, University of Florida, Gainesville, FL, USA; 4 Clinical and Translational Science Institute, University of Florida

## Abstract

OBJECTIVES/SPECIFIC AIMS: Despite aggressive chemotherapy, surgical resection, and radiation therapy, glioblastoma remains almost universally fatal. In a pilot, randomized, and blinded clinical trial, we recently demonstrated that administration of RNA-loaded DC vaccines was associated with significantly improved progression-free and overall survival in patients with glioblastoma (Mitchell *et al*., *Nature*, 2015). Furthermore, clinical outcomes correlated with DC migration to vaccine-site draining lymph nodes measured by Indium-111 labeling of RNA-loaded DCs and SPECT/CT imaging. Although these studies demonstrated that tracking DC migration may be an important clinical biomarker for response to DC vaccination, the complexity and regulatory requirements associated with nuclear labelling to track DC migration limits widespread application of this technique. We have therefore developed RNA-loaded magnetic nanoparticles (RNA-NPs) to enhance DC migration to LNs and track that migration with a widely available imaging modality (i.e., MRI). METHODS/STUDY POPULATION: Cationic liposomes were loaded with iron oxide nanoparticles with or without cholesterol. The resulting nanoparticles were complexed with RNA and used to transfect DCs ex vivo. RNA-NP-loaded DsRed+ DCs were then injected intradermally into mice and tracked noninvasively with T2-weighted 11T MRI before excision and quantification with flow cytometry. RESULTS/ANTICIPATED RESULTS: In vitro experiments demonstrate that iron oxide loading does not reduce RNA-NP-mediated transfection of DCs. Additionally, replacement of cationic lipids with cholesterol increased RNA-NP transfection of the DC2.4 cell line and enhanced the T cell stimulatory capacity of treated bone marrow-derived dendritic cells (BMDCs). Compared to electroporation, RNA-NPs enhanced DC migration to lymph nodes and reduced T2 MRI intensity in DC-bearing lymph nodes. DISCUSSION/SIGNIFICANCE OF IMPACT: This data suggests that iron oxide-loaded RNA-NPs enable noninvasive cell tracking with MRI and enhance DC migration to lymph nodes. We have further shown that inclusion of cholesterol in RNA-NPs augments the stimulatory capacity of transfected DCs. Future work will consider effects of RNA-NPs on antitumor immune responses and the utility of MRI-detected DC migration as a biomarker of vaccine efficacy.

